# Clinical Significance of Skin Toxicity due to EGFR-Targeted Therapies

**DOI:** 10.1155/2009/849051

**Published:** 2009-06-22

**Authors:** Monica Giovannini, Vanesa Gregorc, Carmen Belli, Elisa Roca, Chiara Lazzari, Maria Grazia Viganò, Anna Serafico, Eugenio Villa

**Affiliations:** Oncology Deptartment, San Raffaele Scientific Institute, 60 Olgettina Street, 20132 Milan, Italy

## Abstract

Many small molecules and monoclonal antibodies blocking the activity of Epidermal Growth factor receptor (EGFR) have been developed and have shown clinical activity in patients with non-small cell lung cancer (NSCLC), pancreatic cancer, and colorectal cancer (CRC), and are in clinical development for a range of other solid tumors. The toxicity profile of such agents is characterized by a typical pattern of cutaneous reactions. In this paper we reviewed the current available data regarding the clinical significance of skin reaction due to EGFR targeted agents. We show that skin toxicity can be considered as predictive marker of response to such drugs and that it is not disease specific; however its potential prognostic value is still to be proven.

## 1. Introduction

Receptors with kinase activity, which are involved in the transmission of pleiotropic proliferation signals, seem to be very promising targets for cancer treatments. Many small molecules or monoclonal antibodies that can block the activity of distinct sets of kinases are now available. Agents that target the epidermal growth factor receptor (EGFR) have demonstrated clinical activity in patients with nonsmall cell lung cancer (NSCLC), pancreatic cancer, and colorectal cancer (CRC), and are in clinical development for a range of other solid tumors [1–4]. However, the tolerability profile of EGFR inhibitors (EGFRIs) is impacted by a unique group of cutaneous reactions [5, 6]. Some of these skin events seem to be related to clinical outcomes and survival and could potentially be useful as surrogate markers for treatment efficacy [7]. We review the current available data regarding the clinical significance of skin reaction due to EGFR targeted agents and its correlation with response to such therapies.

**EGFR**

The human epidermal growth factor receptor (HER1/EGFR) is a transmembrane glycoprotein of the tyrosine kinase growth factor family that is expressed in many normal human tissues and several tumors such as colorectal (65–75%), head and neck (90%), and lung (60%–90%) carcinomas [8]. Activation of EGFR by ligands, such as EGF, leads to receptor dimerization and activation of intrinsic tyrosine kinase (TK) activity. This activates downstream signaling pathways, including the mitogen-activated protein kinase (MAPK) and the phosphatidylinositol-3-OH kinase (PI3K/Akt) pathway, modulating gene transcription and protein translation and ultimately stimulating tumor-cell proliferation, migration, adhesion and angiogenesis and inhibiting apoptosis [9]. Overexpression has been correlated to uncontrolled cell growth, proliferation, angiogenesis and metastases. It is a strong prognostic factor as it correlates with increased metastasis, reduced survival, and a poor outcome [10].

**EGFR Targeted Agents: See Table 1**

Two main classes of EGFR targeted agents have been developed so far: monoclonal antibodies (mAb) which block the extracellular domain of the receptor preventing ligand-dependent activation and downstream signalling and small molecule inhibitors (TKI) orally administered, low molecular weight compounds directed against the intracellular tyrosine kinase domain blocking the intracytoplasmic ATP-biding site on the receptor, preventing downstream signal transduction [11].

Cetuximab is a chimeric IgG1 mAb that is currently approved in combination with irinotecan in the EU and USA for EGFR-expressing metastatic CRC in patients who are refractory to irinotecan-based chemotherapy, and as monotherapy in the USA in patients who are intolerant to irinotecan-based chemotherapy. It is also approved for locally or regionally advanced head and neck squamous cell carcinoma (HNSCC) in combination with radiation therapy in the EU and USA, and metastatic or recurrent HNSCC that is refractory to platinum-based therapy, in the USA [10, 12].

The TKI Gefitinib is also currently approved in the USA as a third-line option for patients with NSCLC, but with restrictions. Although this accelerated approval is based on the results of a randomized phase II trial, data from a phase III confirmatory trial failed to show a survival benefit. As a result, the use of gefitinib is at present restricted to patients currently or previously benefiting from it, and to patients enrolled in clinical studies in the USA In addition, it is currently approved for the treatment of inoperable or recurrent NSCLC in Japan and several other Asian countries [13, 14].

Erlotinib, another EGFR TKI, is currently approved in the EU and USA as monotherapy for the treatment of patients with locally advanced or metastatic NSCLC after failure of at least one prior chemotherapy regimen. It is also currently approved, in both regions, for the first-line treatment of patients with locally advanced, unresectable or metastatic pancreatic cancer, in combination with gemcitabine [15, 16].

Bevacizumab is the first vascular endothelial growth factor-targeted agent shown to increase survival in patients receiving first- and second-line intravenous 5-FU-based chemotherapy for the treatment of metastatic colorectal cancer and recently it has been approved also in combination with carboplatin and paclitaxel, for first-line treatment of patients with unresectable, locally advanced, recurrent or metastatic nonsquamous, nonsmall cell lung cancer and in combination with paclitaxel for the treatment of patients who have not received chemotherapy for metastatic HER2-negative breast cancer [17, 18].

Panitumumab, a human IgG2 mAb, is currently approved in the USA for EGFR-expressing, metastatic CRC with disease progression on or following fluoropyrimidine-, oxaliplatin-, and irinotecan-containing chemotherapy regimens [19, 20].

**Most Common Skin Toxicities: See Table 2**

The blockade of the receptor in skin and appendages leads to various cutaneous reactions, occurring, on average, in >50% of patients who receive treatment [21]. EGFRI-associated skin rash appears to be dose dependent [22] and, in general, tends to be more frequent and of higher grade with mAbs than with TKIs [23]. However, the similar spectrum of events observed with both mAbs and TKIs suggests that dermatologic toxicities are likely to be a class effect of these agents. A range of adverse cutaneous reactions with variable severity have been described so far. Xerosis (dry skin), pruritus, nail/periungual alterations (usually manifested as paronychia), regulatory abnormalities of hair growth (usually manifested as alopecia of the scalp and trichomegaly of the eyelashes/hypertrichosis of the face), and telangiectasia (dilatation of capillaries and small blood vessels and hyperpigmentation) have all been observed [24, 25]. The most commonly reported toxicity is a papulopustular reaction. This usually develops on the face and/or upper trunk and, in a majority of cases, peaks in severity during the first 1-2 weeks of therapy, stabilizing during the following weeks. More specifically, the rash commonly develops in the following phases: sensory disturbance with erythema and edema (week 0-1), papulopustular eruption (weeks 1–3), crusting (weeks 3–5), and ending with erythematotelangiectasias (weeks 5–8) [26].

**Pathogenesis of Skin Toxicity**

Mechanisms underlying EGFRI-associated skin toxicities are far to be fully characterized; however, interference with the follicular and interfollicular epidermal-growth signalling pathway is considered critical. Within the epidermis, EGFR plays a critical role, stimulating epidermal growth, inhibiting differentiation, protecting against UV-induced damage, inhibiting inflammation, and accelerating wound healing [27]. EGFR is known to be expressed in epidermal keratinocytes, sebaceous and eccrine glands, and hair follicle epithelium [28], and the greatest expression occurs in proliferating and undifferentiated keratinocytes, which are located in the basal and suprabasal layers of the epidermis and outer root sheath of the hair follicle [29]. Drug-induced inhibition of EGFR is thought to alter keratinocyte proliferation, differentiation, migration, and attachment [30, 31] and this may help to explain the papulopustular reaction and xerosis.

A mixed inflammatory infiltrate surrounding the upper areas of the dermis (especially around follicles), follicular rupture, and epithelial acantholysis have been described in histological specimens taken from the skin of patients with EGFRI-associated rash [32]. In normal skin, phosphorylated EGFR is expressed in the basal and suprabasal layers, and MAPK is observed in the basal layer. Treatment with EGFRIs leads to abolishment of phosphorylated EGFR in all epidermal cells and reduced expression of MAPK. Inhibition of EGFR in basal keratinocytes leads to growth arrest and premature differentiation. This is demonstrated by upregulated expression of cyclin-dependent-kinase inhibitor p27, keratin-1, and signal transducer and activator of transcription-3 in the basal layer, markers of differentiation that are normally only observed within the suprabasal layer [33]. These events lead to release of inflammatory cell chemoattractants that recruit leukocytes which are able to release enzymes resulting in keratinocyte apoptosis, and accumulation of these nonviable cells in the underlying dermis results in additional cutaneous injury, which is thought to account for a majority of symptoms, including tenderness, papulopustules, and periungual inflammation [24]. These changes may also favor bacterial overgrowth, thus exacerbating inflammation, and have also been observed in histological specimens from skin of treated patients. Eventually, a decrease in thickness of the epidermis is observed, demonstrating a thin stratum corneum that lacks its characteristic basketweave configuration (an indication of abnormal differentiation) [33]. Interestingly, it has been shown, in patients who have received radiotherapy prior to EGFRI administration, that areas of skin having undergone prior irradiation tend not to develop a rash during erlotinib therapy [34]. This is thought to be a result of depletion of EGFR-expressing cells or alterations in the microvasculature in the skin covering irradiated areas. Reviewing the existing literature on this topic, we previously observed that in the sequential approach, when radiotherapy is followed by EGFRI, no synergic/additive effect between radiotherapy and EGFRI occurs, as conversely observed with the concomitant approach; such an effect seems to be detrimental, inducing a decrease in expression and activity of EGFR. Moreover this effect seems to be milder if EGFRI is administered immediately after radiotherapy (by 3 weeks) and much stronger when administered after a longer time from radiotherapy [35].

## 2. Correlation between Skin Toxicity and Response to EGFR Targeted Therapies

### 2.1. Cetuximab

In colorectal, pancreatic, and head and neck cancer patients treated with cetuximab a correlation between skin rash and outcome has been clearly described, above all in colorectal cancer setting where cetuximab has been used for a long time as standard treatment option in advanced disease.

**Colorectal Cancer**

In the OPUS study, a phase II randomized trial, 337 patients with untreated EGFR expressing advanced colorectal cancer not respectable with curative intent have been randomized between FOLFOX4 and FOLFOX plus Cetuximab [36]. Median time to rash onset was 21 days. A correlation between response rate and skin toxicity was observed: 13% of patients with no skin toxicity responded versus 42.2% if G1, 53.2% if G2, and 66.7% if G3-4.

In the phase III randomised Crystal Study 1217, EGFR expressing advanced colorectal cancer patients were randomized to FOLFIRI plus Cetuximab versus FOLFIRI [37]. Overall a slight benefit in terms of overall response rate and progression free survival Cetuximab was achieved in the combination arm (*P* = .05); however, greater the skin toxicity higher the progression free survival, despite the lack of data regarding the correlation between rash and overall response rate and survival.

In 2004 Saltz et al. published a phase II open-label clinical trial in metastatic EGFR expressing colorectal cancer treated with Cetuximab after at least one prior line of chemotherapy; 57 patients were enrolled [38]. A clear correlation between outcome, in terms of median survival, and severity of skin toxicity was observed (*P* = .02): 1.9 months if G0 versus 9.5 months if G3 skin toxicity. In the BOND study 329 EGFR expressing advanced colorectal cancer patients after one prior line of treatment were randomized to Cetuximab plus Irinotecan versus Cetuximab [39]. Better response rate and survival were achieved in the combination arm (22.9% versus 10.8%; 8 months versus 6.9 months, resp.). In particular, the greater the skin toxicity the better response and survival, in both arms. In fact, response rates in the 2 arms were 6.3% versus 0% if no skin toxicity, 25.8% versus 12.9% if any grade was considered and 33.6% versus 20% if only G ≥ 2 was considered; all the correlations were statistically highly significant. Any level of EGFR expression seemed to be linked to an equal chance of response to treatment. Regarding this aspect, Saltz et al. reported in JCO that cetuximab shows activity in colorectal cancer patients with tumors that do not express the epidermal growth factor receptor by immunohistochemistry [40]. Sobrero et al. presented at AACR in 2007 the EPIC study, a multicenter, open-label, phase III study in which 1,298 colorectal patients in II line of treatment (after oxaliplatin-based therapy) were randomized to Cetuximab plus Irinotecan versus Irinotecan [41]. A correlation between grade of skin toxicity and outcome was reported: median survival was 5.8 months if G0, 11.7 months if G1-2, and 15.6 months if G3-4. In the NCIC CO.17 phase III randomized study advanced colorectal cancer patients who failed all recommended therapies were randomized to Cetuximab versus best supportive care [42]. Better response rate, progression free survival and survival were achieved in the Cetuximab arm; skin toxicity was correlated to better outcome: overall survival was 2.6 months if G0, 4.8 if G1 and 8.4 if G2 (*P* < .001).

The EVEREST study is a phase I/II study in which 166 Irinotecan refractory advanced colorectal cancer patients after 3 weeks of treatment with Irinotecan plus Cetuximab were randomized on the basis of skin toxicity: if G < 2 patients were randomized to classic Cetuximab dose versus Cetuximab dose escalation performed every 2 weeks until skin toxicity grade > 2 [43]. Dose escalation arm showed better response rate, disease control rate, duration of response, progression free survival and median survival compared to classic schedule.

**Pancreatic Cancer**

Xiong et al. published in JCO a phase II study with the combination of Gemcitabine plus Cetuximab as first line treatment in 41 advanced pancreatic cancer patients [44]. Higher skin toxicity was associated with better outcome in terms of median survival (2.3 months if G0, 5.7 if G1, 8 if G2 and 13.9 if G3; *P* = .0007).

**Head and Neck Cancer**

A retrospective study was conducted in 211 patients treated with Cetuximab and radiotherapy after failure with Cisplatinum-based chemotherapy [45]. Better outcome in terms of survival was achieved in “the prominent” rash group (Grade 2–4) compared to “the minimal” rash group (G0-1): median survival was 56.7 months and 24.4 months, respectively, 3-year survival rate was 65% versus 42%, respectively.

### 2.2. Gefitinib

In nonsmall cell lung cancer and head & neck cancer patients treated with gefitinib a correlation between skin rash and outcome has been reported.

**Non Small Cell Lung Cancer**

The IDEAL trials 1 and 2 were carried on to explore safety and efficacy of Gefitinib (250 mg/day versus 500 mg/day) in pretreated advanced non small cell lung cancer patients (as 1st and 2nd line in IDEAL 1 and as 2nd and beyond in IDEAL 2) [46, 47]. No difference was registered between the 2 dosages in both studies in terms of outcome. The occurrence of early onset skin toxicity in patients who survived at least 28 days was analysed retrospectively: 67% of patients who ultimately responded did not experience skin toxicity by day 24; 25% of patients who ultimately responded did not have skin toxicity by day 28. There was no statistically significant difference in the objective response rate between those with or without early onset skin toxicity. In the Gefitinib Expanded Access Program patients showing skin toxicity performed better in terms of survival compared to those without skin toxicity (10.8 versus 4 months, *P* = .0001) [48].

**Head and Neck Cancer**

Gefitinib was administered to 52 pretreated Head and Neck cancer patients in a phase II trial; skin toxicity was associated with better median survival (11.1 versus 5.3 months, *P* = .001) compared to patients without skin toxicity [49].

### 2.3. Erlotinib

Also in non small cell lung cancer, pancreatic and head & neck cancer skin rash seems to be associated with better outcome has been reported in patients treated with erlotinib.

**Non Small Cell Lung Cancer**

In the BR21 trial advanced nonsmall cell lung cancer patients after one or two lines of therapy were randomized to erlotinib versus placebo [50]. In 485 patients in erlotinib arm skin rash occurred in 75% [G1-2 66%, G3 8%, G4 < 1%), median time of onset was 8 days (1–113), 10% of patients needed dose reduction because of skin toxicity, in 7% of patients dose reduction due to skin toxicity lasted > 7 days, in 3% of patients > 14 days. Response rate was higher in patients with at least G2 skin toxicity (complete/partial response was 13% if G ≥ 2 versus 10% if G1 versus 0% if G0; complete/partial response/stable disease was 60% if G ≥ 2 versus 50% if G1 versus 16% if G0; all the correlations were statistically significant). Similarly median survival was 11.1 months if G ≥ 2 versus 7.1 months if G1 versus 3.3 months if G0 (*P* < .001) and progression free survival was 4 months if G ≥ 2 versus 3.2 months if G1 versus 1.7 months if G0 (*P* < .001). In the multivariate analysis gender, age, race, histology, performance status, smoking status, prior weight loss, EGFR status measured by IHC, gene copy number by FISH, mutational status for EGFR and KRAS and time from initial diagnosis were included. A correlation between skin toxicity and progression free survival was observed (HR 0.51 if G1 *P* < .001, HR 0.35 if G ≥ 2 *P* < .001); similarly a strong correlation between skin toxicity and survival (HR 0.51 if G1 *P* < .001, HR 0.34 if G ≥ 2 *P* < .001).

**Pancreatic Cancer**

In PA.3 trial 254 patients with locally advanced or metastatic pancreatic cancer were randomized to erlotinib plus gemcitabine versus gemcitabine alone [50]. Skin rash occurred in 71% (G 1-2 66%, G3 3%, G4 2%); median time of onset was 10 days (10–44 days), 2% of patients needed dose reduction because of skin toxicity, in 5% of patients dose reduction due to skin toxicity lasted > 7 days, in 3% of patients the interruption was permanent. Poor performance status was inversely correlated to skin toxicity incidence (*P* = .01). Response rate was higher in patients with at least G2 skin toxicity (complete/partial response was 15% if G ≥ 2 versus 7% if G1 versus 6% if G0; complete/partial response/stable disease was 74% if G ≥ 2 versus 58% if G1 versus 49% if G0; all the correlations were statistically significant). Similarly median survival was 10.8 months if G ≥ 2 versus 5.7 months if G1 versus 5.4 months if G0 (*P* < .001, *P* = .5, *P* < .001) and progression free survival was 6.5 months if G ≥ 2 versus 3.6 months if G1 versus 3.1 months if G0 (*P* < .001). In the multivariate analysis gender, age, race, performance status, prior chemotherapy, baseline pain score, baseline albumin were included. A correlation between skin toxicity and progression free survival if G ≥ 2 but not for milder grade was observed (HR 0.43 if G ≥ 2 versus G0 *P* < .001, HR 0.98 if G1 *P* = .881); similarly a strong correlation between skin toxicity and survival if G ≥ 2 but not for milder grade (HR 0.46 if G ≥ 2 versus G0 *P* < .001, HR 0.93 if G1 *P* = .66).

**Head and Neck Cancer**

Erlotinib was administered to 199 locally advanced and advanced head and neck treated with no more than one prior therapy cancer in a phase II trial [51]. Median survival was higher if skin toxicity G ≥ 2 occurred (224 days versus 120 days *P* = .045) but not in patients with G1 skin toxicity (153 days *P* = .15).

### 2.4. Panitumumab

Very few data are currently available regarding the correlation between skin toxicity and response to treatment with panitumumab. A multicenter randomized phase III trial was conducted in a population of heavily pretreated patients with metastatic CRC [52]. In total, 463 patients with 1% or more EGFR expression in the tumor, measurable disease, and radiological evidence of disease progression during or within 6 months of most recent chemotherapy were randomized to receive either 6 mg/kg panitumumab every 2 weeks plus best supportive care (BSC; *n* = 231) or BSC alone (*n* = 232). Objective response rates favored panitumumab plus BSC (10%) over BSC alone (0%; *P* < .0001). The median time to response was 7.9 weeks and the median duration of response was 17.0 weeks. A further 64 (28%) and 24 (10%) patients in the panitumumab and BSC arms, respectively, experienced stable disease as the best response. Similar activity was reported in patients enrolled in the cross-over study. No difference in overall survival was seen between the two arms, probably because of the large number of patients assigned to BSC who crossed over to the addition of panitumumab. In an exploratory analysis, both progression-free survival and overall survival rates were greater in patients with grade 2–4 skin toxic events than in those with grade 1 events. Nineteen (86%) of 22 responders had skin toxic events of grade 2 or 3, whilst the remaining 3 (14%) responders experienced grade 1 skin toxic events.

### 2.5. Bevacizumab

Although exfoliative dermatitis has been described as a side effect in 19% of patients, skin rash (type unspecified) has rarely been described in patients following infusion of bevacizumab. Saiff et al. have recently reported the first patient with colon cancer manifesting a correlation between rash and a positive drug response with bevacizumab [53]. A 49-year old male with T3 N1 M1 rectal carcinoma received modified FOLFOX-6/bevacizumab, which he tolerated very well except for grade 2 skin rash related to bevacizumab. The rash continued to progress as the serum carcinoembryonic antigen decreased significantly. Computed tomography and positron emission tomography scan confirmed response to FOLFOX/bevacizumab. Therefore this rash was linked to bevacizumab administration and correlated with response to therapy.

## 3. Conclusions

Skin toxicity is peculiarly associated with EGFR targeted agents, both monoclonal antibodies, such as cetuximab, and small molecules Tyrosin Kinase Inhibitors such as gefitinib and erlotinib. Such analysis is challenging as no specific grading for skin toxicity is currently available and its evaluation is often “clinician dependent.” Despite this potential bias an association between skin toxicity and outcome to treatment has been observed in most cases. It seems not to be disease specific as it occurs in patients treated with EGFR targeted therapies for colorectal cancer, pancreatic cancer, head & neck cancer and non small cell lung cancer. Moreover and more important its occurrence is clearly predictive of treatment outcome and its severity is expression of the benefit gain from such therapies, independently from EGFR targeted agent, line of treatment and chemotherapy associated with EGFR inhibitors. Hence, skin toxicity can be considered a predictive marker, surrogate of activity and efficacy of EGFR targeted therapies. In fact, skin toxicity can be seen as a matter of pharmacodynamics; it can be considered a marker of systemic drug concentration and consequently of drug activity, independently from tumor histology. Moreover the difference in severity of skin toxicity due to monocloclonal antibodies or small molecules can be explained by different pharmacodynamics factors, such as transporter proteins, for example, ABCG2 (55), which can interfere with intestinal absorption of TKI. Its potential prognostic value is still to be proven. Prior identification of patients more likely to develop skin toxicity and thus to benefit from EGFR targeted therapies remains a key issue. Moreover, a specific scale measuring not only rash intensity but also the impact on aesthetics and functionality has to be developed in order to help physicians in the decision making process and patient toxicity management. Clinicians have been getting more and more used to managing skin toxicity due to EGFR targeted agents; however it remains a big issue for the patients as it can interfere dramatically with their social life, also because of the lack of standard treatment guidelines. 

## Figures and Tables

**Table 1:** EGFR targeted agents.

Agent	Class	Indication	Dose
Erlotinib	TKI	- Locally advanced or metastatic NSCLC after at least one prior chemotherapy regimen	100–150 mg/day cancer
		- Locally advanced or metastatic pancreatic cancer in combination with gemcitabine	

Gefitinib	TKI	- As single agent Locally advanced or metastatic NSCLC after at least platinum based and docetaxel chemotherapy regimen (only in the USA)	250 mg/day

Catuximab	mAb	- Locally or regionally advanced squamous cell carcinoma of head and neck in combination with radiotherapy	400 mg/m^2^ initial dose followed by 250 mg/m^2^ weekly
		- As single agent for recurrent or metastatic squamous cell carcinoma of head and neck after failure of platinum-based chemotherapy	
		- As single agent in EGFR-expressing metastatic colorectal carcinoma in case of intolerance to irinotecan-based chemotherapy	
		- In combination with irinotecan in EGFR-expressing metastatic colorectal carcinoma in patients refractory to irinotecan-based chemotherapy	

Panitumumab	mAb	- In EGFR-expressing metastatic colorectal carcinoma in patients in progression on or following fliuoropyrimidine-, oxaliplatin-, and irinotecan-based chemotherapy	6 mg/kg iv every 14 days

Bevacizumab	mAb	- Advanced colorectal cancer patients receiving first- and second-line intravenous 5-FU-based chemotherapy for the treatment	5–15 mg/kg/2 weeks
		- In combination with carboplatin and paclitaxel, for first-line treatment of patients with unresectable, locally advanced, recurrent or metastatic nonsquamous, nonsmall cell lung cancer	
		- In combination with paclitaxel for the treatment of patients who have not received chemotherapy for metastatic HER2-negative breast cancer.	

**Table 2:** Most common skin toxicities.

Adverse event	Frequency	Description
Rash	60–80%	Monomorphous erythematous maculopapular, follicular, or pustolar lesions which may be associated with pruritus/tenderness
Paronychia and fissuring	6–12%	Painful periungual granulation-type or friable pyogenic granuloma-like changes, associated with erythema, swelling, and fissuring of lateral nailfolds and/or distal finger tufts
Hair changes	5–6%	Alopecia and curlier, finer and more brittle hair on scalp and extremities; trychomegalia and curling of eyebrows and hypertrichosis of the face
Dry skin	4–35%	Diffuse fine scaling
Mucositis	2–36%	Mild to moderate mucositis, stomatitis, and aphthous ulcers
Hypersensitivity reactions	2–3%	Flushicg, urticaria, and anaphylaxis
